# Immunological consequences of delayed exposure to hepatitis A virus: evidence consistent with CD8^+^ T-cell–mediated immunopathology

**DOI:** 10.1186/s12879-026-13193-x

**Published:** 2026-04-11

**Authors:** Peter Asaga Mac, Vijeesh Kadukkatti, Mathew K. Brijil, Philomena Airiohuodion

**Affiliations:** 1 Institute for Infection Prevention and Control, Medical Center, Faculty of Medicine, University of Freiburg, Breisacher Str. 115B, 79014 Freiburg, Germany; 2 Healthy Living Spaces lab, Institute for Occupational, Social and Environmental Medicine, Medical Faculty, RWTH Aachen University, Pauwelsstr. 30, 52074 Aachen, Germany; 3 Aster MIMS Hospital, Kerala 670621 Kannur, India; 4 WHO/TDR Clinical Research Fellowship Programme, Geneva, Switzerland

**Keywords:** Hepatitis A virus, Epidemiological transition, Urbanisation, Immune-mediated liver injury, CD8 + T-cell response, Vaccination policy

## Abstract

**Background:**

Hepatitis A virus (HAV) infection has traditionally occurred during early childhood in high-endemic settings but is increasingly affecting adolescents and adults in regions undergoing epidemiological transition, where improvements in sanitation delay primary exposure. Adult HAV infection is associated with more severe clinical disease, and prior work has implicated CD8 + cytotoxic T lymphocyte responses and cytokine-driven bystander immune activation in hepatocyte injury; however, the immunological correlates underlying age-dependent severity in populations undergoing active epidemiological transition remain incompletely characterised.

**Methods:**

We analysed statewide surveillance data from Kerala, India (2020–2024), comprising 34,118 confirmed or probable HAV cases, to characterise temporal, seasonal, and geographic trends. Within this framework, we conducted an immunological substudy of 180 patients with confirmed acute HAV infection, stratified by age at infection used as an operational proxy for early (< 18 years) versus delayed (≥ 18 years) primary exposure in a population where improvements in sanitation have progressively shifted first infection to later ages. Clinical severity, hospitalisation, liver biochemistry, peripheral CD8 + T-cell responses (IFN-γ ELISPOT), plasma cytokines, and viral load were assessed. Multivariable logistic regression and sensitivity analyses were used to identify factors associated with moderate-to-severe disease.

**Results:**

Surveillance data demonstrated a marked increase in reported HAV cases after 2021, with pronounced post-monsoon seasonality and geographic clustering. In the immunological cohort, individuals infected at ≥ 18 years had significantly higher odds of moderate-to-severe disease compared with those infected earlier (adjusted odds ratio 8.42, 95% CI 3.21–22.08). Moderate-to-severe disease occurred in 69.5% of participants with delayed exposure compared with 7.7% of those with early exposure. Delayed exposure was associated with stronger peripheral CD8 + T-cell IFN-γ responses and higher plasma IFN-γ and TNF-α concentrations, alongside higher aminotransferase levels and increased hospitalisation rates. Viral load did not differ significantly between age groups. Findings were robust across multiple sensitivity analyses.

**Conclusions:**

In this transitioning setting, adult primary HAV infection is associated with increased clinical severity and heightened peripheral cellular immune responses, independent of viral load. These peripheral immune findings are consistent with an immune-mediated contribution to liver injury during adult HAV infection. The findings support consideration of public health strategies aimed at preventing delayed exposure, including prioritisation of childhood hepatitis A vaccination in regions experiencing similar epidemiological transitions.

**Graphical Abstract:**

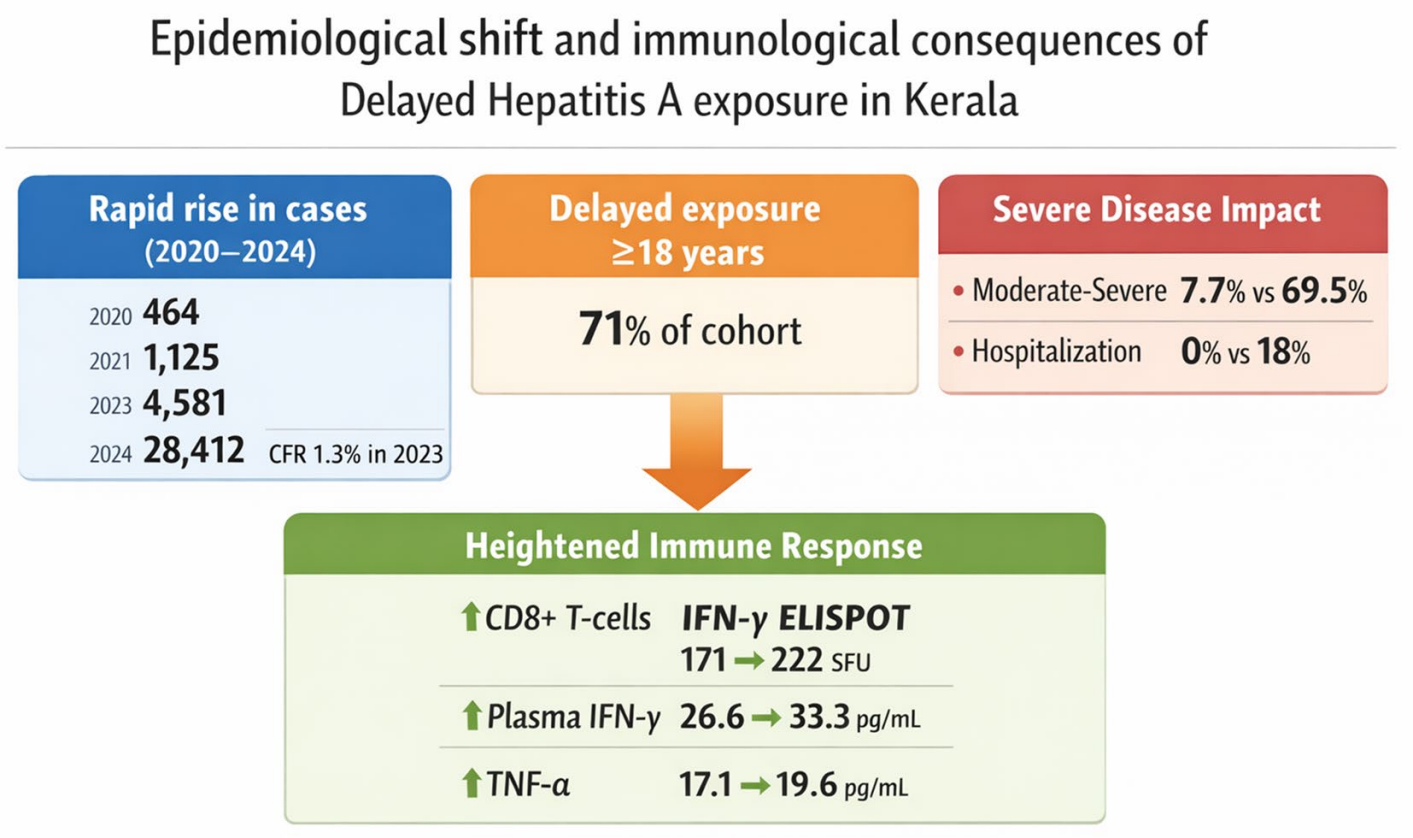

**Supplementary Information:**

The online version contains supplementary material available at 10.1186/s12879-026-13193-x.

## Introduction

Hepatitis A virus (HAV) infection remains an important cause of acute viral hepatitis worldwide, despite the long-standing availability of safe and highly effective vaccines. Safe and highly effective inactivated HAV vaccines induce seroconversion in over 95% of recipients following a two-dose schedule, conferring durable protective immunity, and have substantially reduced disease burden in settings where vaccination has been implemented [1, 2]. Recent global assessments indicate that HAV continues to account for a substantial burden of infection and preventable mortality, particularly in regions undergoing rapid socioeconomic change [3–5]. Unlike hepatitis B and C viruses, HAV does not establish chronic infection; nevertheless, its public health impact persists through recurrent outbreaks, healthcare system strain, and pronounced age-related differences in clinical severity [6, 7].

The epidemiology of hepatitis A is closely linked to access to safe water, sanitation, and hygiene. In settings of high endemicity, exposure typically occurs in early childhood, resulting in asymptomatic or mild infection and durable lifelong immunity [8, 9]. As living conditions improve and transmission intensity declines, populations transition towards intermediate endemicity, characterised by reduced childhood exposure and the accumulation of susceptible adolescents and adults [10–12]. This transition produces a paradoxical increase in clinically apparent disease, as primary HAV infection in adulthood is associated with a far higher likelihood of jaundice, prolonged illness, hospitalisation, and death [13–15]. These shifts in exposure patterns have been widely documented in regions undergoing rapid urbanisation and socioeconomic development, including in South Korea, where a marked increase in young adult infections followed improvements in childhood sanitation [16], and in India, where population-based seroprevalence studies have demonstrated declining childhood immunity across multiple regions [17].

Age at primary infection is among the strongest determinants of clinical outcome in hepatitis A. While young children frequently experience subclinical infection, most adults develop symptomatic disease, with severity and case fatality rising progressively with age [18–20]. This pattern has been consistently observed across diverse geographic and socioeconomic contexts, including in recent outbreaks in regions previously considered low risk [21–23]. Importantly, differences in clinical severity are not explained by viral genotype or replication kinetics, pointing instead to host immune responses as the principal drivers of hepatocellular injury [24, 25].

A growing body of evidence supports an immune-mediated model of liver injury in hepatitis A. HAV replication is largely non-cytopathic; hepatocyte damage arises predominantly from host immune responses rather than from direct viral cytotoxicity. The onset of hepatic inflammation coincides temporally with the emergence of adaptive immune responses rather than peak viraemia [26, 27]. CD8 + cytotoxic T lymphocytes are central effectors of this process, mediating hepatocyte injury through perforin–granzyme pathways and the secretion of proinflammatory cytokines, particularly interferon-γ (IFN-γ) and tumour necrosis factor-α (TNF-α) [28–30]. Both human studies and experimental models have demonstrated that the magnitude of these cellular immune responses correlates more closely with biochemical markers of liver injury than with viral load [31, 32].

More recent work has further refined this paradigm by demonstrating that immune-mediated pathology in hepatitis A extends beyond virus-specific responses. Cytokine-driven bystander activation of memory CD8 + T cells specific to unrelated pathogens can occur during acute infection, conferring innate-like cytotoxic potential and amplifying liver injury [33–35]. This phenomenon, which has been proposed as a contributing mechanism in hepatitis A immunopathology but was not directly assessed in the present study, appears to be more prominent in adults, who possess a larger and more diverse memory T-cell compartment than children, providing a biologically plausible explanation for the striking age dependence of disease severity [36, 37].

India illustrates the public health consequences of hepatitis A epidemiological transition, with marked regional heterogeneity. The southern state of Kerala, despite advanced health and social indicators, has experienced repeated hepatitis A outbreaks increasingly affecting adolescents and adults [38–41]. Seroprevalence studies indicate reduced population-level immunity due to decreased childhood exposure, while recent surveillance data demonstrate a sharp increase in reported cases, rising hospitalisation rates, and increasing case fatality [42–45]. These trends underscore the urgency of understanding the immunological mechanisms underpinning disease severity in this transitional context. However, while prior studies have described age-related differences in clinical severity and immune activation, most have focused on outbreak settings or single clinical cohorts and have not integrated large-scale epidemiological surveillance with detailed immunological profiling in populations undergoing active epidemiological transition.

We therefore hypothesised that delayed primary exposure to HAV is associated with augmented cellular immune responses and heightened inflammatory signalling, resulting in more severe hepatocellular injury, independent of viral burden. To test this hypothesis, we conducted an integrated analysis combining statewide surveillance data from Kerala with a nested prospective immunological study of patients with acute hepatitis A. By situating mechanistic immune findings within a rapidly evolving epidemiological landscape, this study seeks to provide a coherent account of the clinical and immunopathological consequences of hepatitis A epidemiological transition, with direct implications for vaccination policy and disease prevention strategies.

## Methods

### Study design and overview

This study employed an integrated observational design comprising two complementary components: a population-level analysis of hepatitis A surveillance data from the state of Kerala, India, and a nested prospective immunological study of patients with acute hepatitis A. This combined approach was designed to situate individual-level immunological findings within the broader epidemiological context of a population undergoing active transition in hepatitis A endemicity.

The surveillance analysis covered the period from January 2020 to December 2024. The immunological substudy was conducted concurrently and enrolled patients presenting with acute hepatitis A from healthcare facilities distributed across all districts of Kerala (Supplementary S1). Samples were collected cross-sectionally at the time of presentation, and each participant contributed a single sample for analysis. No repeated measures were included, and all samples analysed were derived from unique individuals.

### Epidemiological surveillance data

Statewide hepatitis A surveillance data were obtained from the Integrated Disease Surveillance Programme (IDSP), Directorate of Health Services, Government of Kerala, through formal data-sharing agreements. The IDSP conducts syndromic and laboratory-based surveillance for notifiable communicable diseases across public health facilities and selected private institutions, with weekly reporting at the district level.

Confirmed hepatitis A was defined as laboratory detection of anti–hepatitis A virus (HAV) immunoglobulin M (IgM) antibodies using validated enzyme immunoassay or chemiluminescent immunoassay platforms, in accordance with national communicable disease surveillance guidelines used by the IDSP [44]. Probable cases were defined by compatible clinical illness, including acute onset of jaundice or elevated aminotransferases, in individuals with epidemiological linkage to confirmed cases or recognised outbreaks. District-level and temporal data were extracted where available, and annual case counts, trends, and case fatality rates were calculated descriptively.

## Immunological substudy population

Patients presenting with symptoms consistent with acute viral hepatitis were consecutively screened for inclusion at participating government medical colleges, district hospitals, and selected primary health centres. Potential participants were identified through laboratory notification of positive anti-HAV IgM results.

Eligibility criteria included age ≥ 1 year, laboratory-confirmed acute HAV infection (anti-HAV IgM positive), symptom duration of 21 days or less at enrolment, and residence in Kerala for at least 12 months prior to illness onset. Exclusion criteria comprised serological or molecular evidence of co-infection with hepatitis B virus, hepatitis C virus, or hepatitis E virus; known chronic liver disease; immunocompromising conditions; current or recent use of immunosuppressive medications; pregnancy (excluded because the immunological adaptations of pregnancy—including a shift towards Th2-predominant cytokine profiles and attenuated cellular immune responses could confound interpretation of the T-cell and cytokine parameters central to this study; ethical considerations regarding the additional burden of research procedures in pregnant women were also taken into account); and documented prior hepatitis A vaccination with confirmed seroconversion before the current illness.

### Clinical and laboratory assessments

Demographic and clinical data were collected at enrolment using standardised case report forms and included age, biological sex, district of residence, symptom duration, comorbidities, and vaccination history. Laboratory assessments performed at presentation included serum ALT, AST, total and direct bilirubin, alkaline phosphatase, gamma-glutamyl transferase, albumin, and coagulation parameters.

Acute HAV infection was confirmed using a chemiluminescent immunoassay for anti-HAV IgM antibodies (ARCHITECT HAVAb-IgM; Abbott Diagnostics), with a signal-to-cutoff ratio ≥ 1.0 considered positive. Anti-HAV IgM is a well-established marker of recent infection, typically becoming detectable within 5–10 days of symptom onset and persisting for 3–6 months [46]. Although anti-HAV IgM in the setting of acute clinical illness is the accepted diagnostic standard, IgG avidity testing was not performed. While IgG avidity is an established tool for certain viral infections such as cytomegalovirus, it has not been incorporated into standard diagnostic practice for hepatitis A—an infection that does not establish latency or undergo reactivation. Nonetheless, rare instances of persistent or recrudescent IgM cannot be entirely excluded. Anti-HAV IgG antibodies were quantified using the ARCHITECT HAVAb-IgG assay (Abbott Diagnostics).

### Immunological assays

Peripheral blood mononuclear cells (PBMCs) were isolated from heparinised venous blood by density gradient centrifugation using Ficoll-Paque PLUS within four hours of collection. Cell viability was assessed by trypan blue exclusion, and samples with viability below 90% were excluded from immunological analyses (*n* = 6 excluded). This threshold was selected to optimise ELISPOT assay performance, as recommended by ELISPOT harmonisation guidelines, given the assay’s sensitivity to dead-cell artefacts and background noise [Janetzki et al. 2009]. Fresh PBMCs were used for immediate assays, and aliquots were cryopreserved in fetal bovine serum containing 10% dimethyl sulfoxide for batch analysis.

HAV-specific CD8 + T-cell responses were quantified using IFN-γ enzyme-linked immunospot assays (Human IFN-γ ELISpotBASIC kit; Mabtech AB). Briefly, 96-well PVDF-bottomed plates were pre-coated with anti-IFN-γ monoclonal antibody overnight at 4 °C. After washing, 2 × 10⁵ PBMCs per well were stimulated with overlapping 15-mer peptide pools spanning the complete HAV polyprotein at a final concentration of 1 µg/mL per peptide for 18–20 h at 37 °C. Phytohaemagglutinin was used as a positive control. Negative control wells contained PBMCs in medium alone. Spot-forming units were enumerated using an automated ELISPOT reader and expressed per 10⁶ PBMCs after subtraction of background responses.

Plasma concentrations of IFN-γ, TNF-α, and IL-6 were measured using a multiplex electrochemiluminescent immunoassay (V-PLEX Proinflammatory Panel 1 Human Kit; Meso Scale Discovery). These cytokines were selected a priori: IFN-γ and TNF-α are established effector cytokines of Th1/CD8 + T-cell responses implicated in HAV-related hepatocyte injury [28–30], while IL-6 was included to assess the contribution of the broader acute-phase inflammatory response. This focused panel does not capture the full breadth of the inflammatory milieu, and additional mediators—including IL-10, IL-18, and chemokines—may contribute to disease pathogenesis. All samples were analysed in duplicate, and the mean value of the two measurements was used for statistical analysis.

### Statistical analysis

Surveillance data were analysed descriptively to assess temporal trends, geographic distribution, and seasonality. For the immunological substudy, distributional characteristics were assessed primarily by visual inspection of histograms and quantile–quantile plots, supplemented by the Shapiro–Wilk test as a secondary adjunct. This combined approach was adopted because the Shapiro–Wilk test may flag trivial departures from normality as statistically significant in larger samples (*n* > 50), even when the data are approximately normally distributed for practical purposes. Where distributional assessment was equivocal, both parametric and non-parametric tests were applied and results compared to ensure robustness. Categorical variables were summarised as counts and percentages.

Between-group comparisons were performed using Student’s t-test or the Mann–Whitney U test for continuous variables and the χ² test or Fisher’s exact test for categorical variables, as appropriate. Correlations were assessed using Spearman’s rank correlation coefficient. Multivariable logistic regression was used to evaluate factors independently associated with moderate-to-severe disease. Statistical analyses were conducted using R (version 4.3.1) and Python (version 3.11.0). A two-sided p value of less than 0.05 was considered statistically significant.

### Multivariable modelling

Multivariable logistic regression was used to identify independent predictors of moderate-to-severe hepatitis A. The primary exposure of interest was delayed primary exposure (≥ 18 years). Covariates were selected a priori based on biological plausibility and included biological sex, comorbidity status, symptom duration at enrolment, HAV-specific CD8 + T-cell IFN-γ ELISPOT responses, plasma IFN-γ concentrations, and plasma TNF-α concentrations. Continuous immunological variables were modelled per prespecified clinically meaningful increments. Adjusted odds ratios (aORs) with 95% confidence intervals (CIs) were reported. The final model demonstrated excellent discrimination (AUC-ROC 0.89, 95% CI 0.84–0.94) and good calibration (Hosmer–Lemeshow *p* = 0.42). Model assumptions were assessed using standard diagnostic procedures [47].

### Sensitivity and robustness analyses

Prespecified sensitivity analyses were conducted to assess the robustness of the association between delayed primary exposure and moderate-to-severe disease. These included alternative age thresholds (12–30 years), exclusion of participants with borderline anti-HAV IgM values, exclusion of participants with comorbidities, exclusion of children younger than 5 years, and exclusion of statistical outliers. Complete case analyses were performed. Subgroup analyses were conducted by biological sex and area of residence, and effect modification was assessed using multiplicative interaction terms. E-values were calculated for the primary exposure.

### Ethical considerations

The study protocol was approved by the Institutional Ethics Committee of Government Medical College, Thiruvananthapuram (IEC No. 01/32/2020/MCT), with reciprocal approval from the Ethics Committee of the Faculty of Medicine, University of Freiburg. Written informed consent was obtained from all participants or their legal guardians. All procedures were conducted in accordance with the Declaration of Helsinki (2013 revision).

## Results

### Epidemiological surveillance findings

Between January 2020 and December 2024, a total of 34,118 confirmed or probable hepatitis A cases were reported to the IDSP from Kerala (Table 1). Note that absolute case numbers may partly or substantially reflect increased testing and surveillance capacity; in the absence of denominator data on total tests performed, trends cannot be attributed solely to changes in transmission.

**Table 1:** Annual hepatitis A surveillance data from Kerala, India, 2020–2024

Year	Confirmed	Probable	Total	Deaths	CFR (%)
2020	464	NR	464+	2	0.43
2021	114	NR	114+	0	0.00
2022	231	894	1,125	2	0.87
2023	1,073	3,508	4,581	14	1.30
2024	7,967	20,445	28,412	89	1.12
**Total**	**9**,**849**	**24**,**847+**	**34**,**118+**	**107**	**1.09**

CFR, case fatality rate. NR, not separately reported; data for 2020–2021 did not distinguish probable from confirmed cases. Source: Integrated Disease Surveillance Programme, State Surveillance Unit, Directorate of Health Services, Government of Kerala

**Fig. 1:**
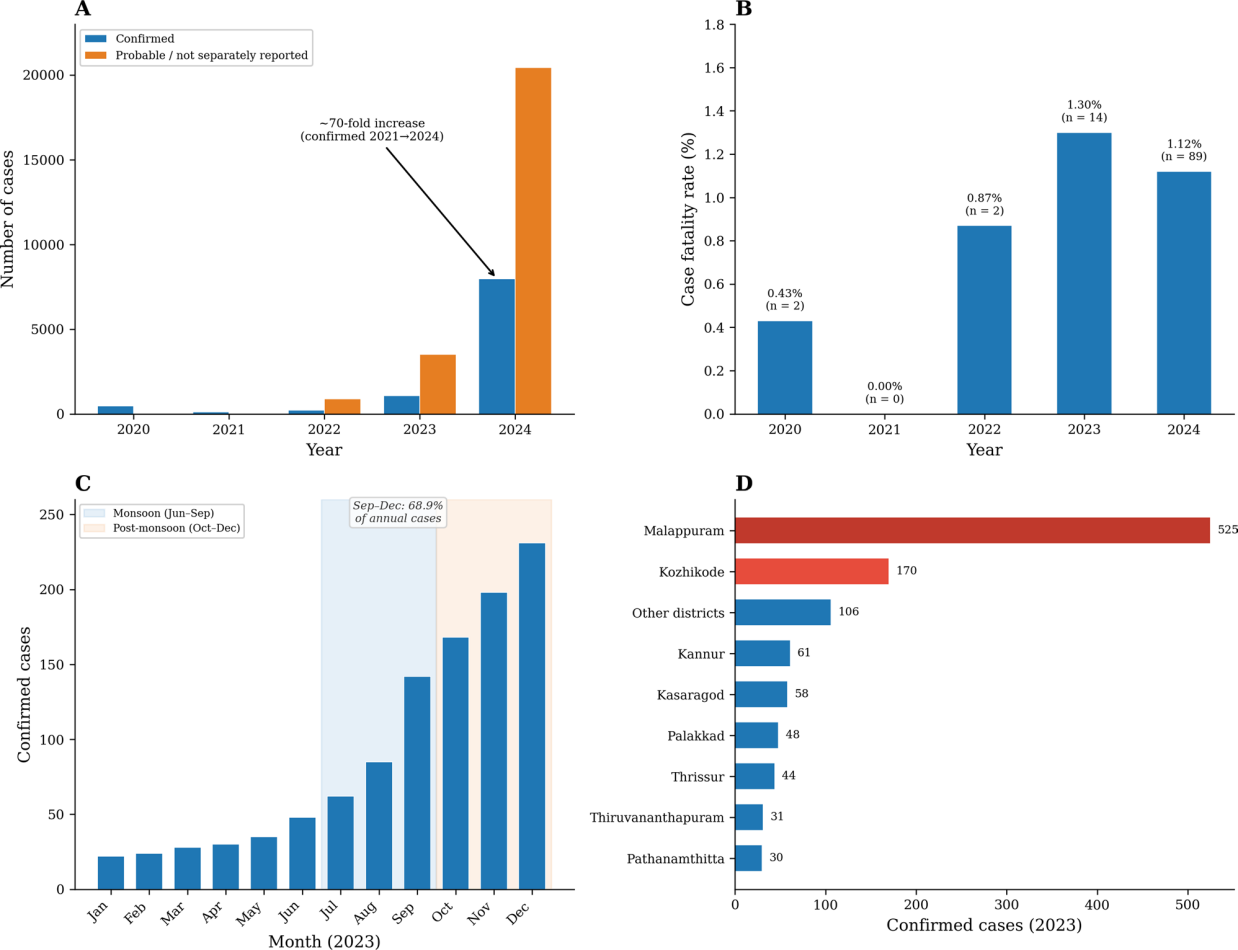
Epidemiological trends in hepatitis A in Kerala, 2020–2024. (**A**) Annual confirmed and probable hepatitis A cases; note that absolute case numbers may partly or substantially reflect increased testing and surveillance capacity, and in the absence of denominator data on total tests performed, trends cannot be attributed solely to changes in transmission. (**B**) Case fatality rates by year with absolute death counts indicated. (**C**) Monthly distribution of confirmed cases in 2023 showing monsoon and post-monsoon seasonality. (**D**) District-wise distribution of confirmed cases in 2023

Confirmed cases declined from 464 in 2020 to 114 in 2021, likely reflecting reduced healthcare utilisation during pandemic-related restrictions. From 2022 onwards, confirmed case numbers rose sharply, increasing to 231 in 2022, 1,073 in 2023, and 7,967 in 2024, representing an approximately 70-fold increase from the 2021 nadir. When probable cases were included, the total burden reached 28,412 cases in 2024 alone (Table 1; Fig. 1).

Case fatality rates among confirmed cases were 0.43% in 2020, 0% in 2021, 0.87% in 2022, 1.30% in 2023, and 1.12% in 2024. Over the five-year period, 107 deaths were recorded, yielding an overall case fatality rate of 1.09% (Fig. 1B).

District-level analysis for 2023 revealed marked geographic heterogeneity (Table 2; Fig. 1D). Malappuram district alone accounted for nearly half of confirmed cases. Similar patterns were observed in subsequent years (Supplementary Table S1). Seasonal analysis demonstrated a consistent increase in case counts during the monsoon and post-monsoon periods (Supplementary Figure S2). In 2023, 68.9% of confirmed cases occurred between September and December (Fig. 1C).

**Table 2:** District-wise distribution of confirmed hepatitis A cases, Kerala, 2023

District	Confirmed cases	Deaths	CFR (%)
Malappuram	525	5	0.95
Kozhikode	170	4	2.35
Kannur	61	3	4.92
Kasaragod	58	0	0.00
Palakkad	48	1	2.08
Thrissur	44	0	0.00
Thiruvananthapuram	31	0	0.00
Pathanamthitta	30	0	0.00
Other districts	106	1	0.94
**Total**	**1**,**073**	**14**	**1.30**

CFR, case fatality rate. Other districts include Kollam, Alappuzha, Kottayam, Idukki, Ernakulam, and Wayanad

### Characteristics of the immunological substudy population

A total of 180 patients with confirmed acute hepatitis A were enrolled (age range 4–58 years; 92 [51.1%] male). Of these, 52 participants (28.9%) were classified as having early exposure (< 18 years; median age 12, IQR 10–15; 28 [53.8%] male), and 128 (71.1%) as having delayed exposure (≥ 18 years; median age 30, IQR 25–37; 64 [50.0%] male). Demographic and baseline characteristics are summarised in Table 3.

**Table 3:** Demographic and baseline characteristics of the immunological substudy population

Characteristic	Early exposure (*n* = 52)	Delayed exposure (*n* = 128)	*p*-value
Age, years, median (IQR)	12 (10–15)	30 (25–37)	< 0.001
Male biological sex, n (%)	28 (53.8)	64 (50.0)	0.64
Prior HAV vaccination, n (%)	10 (19.2)	25 (19.5)	0.96
Symptom duration, days	7.2 ± 3.1	8.1 ± 3.8	0.13
Anti-HAV IgM index (S/CO)	5.43 ± 3.20	5.05 ± 2.77	0.43
Anti-HAV IgG titre (AU/mL)	61.5 ± 21.7	73.5 ± 21.5	0.001

Data are presented as n (%), mean ± SD, or median (IQR). HAV, hepatitis A virus; IgG, immunoglobulin G; IgM, immunoglobulin M; IQR, interquartile range; S/CO, signal-to-cutoff ratio; SD, standard deviation. Between-group comparisons: Mann–Whitney U test (age), Student’s t-test (symptom duration, IgM index, IgG titre), χ² test (biological sex, vaccination history)

### Clinical outcomes and disease severity

**Fig. 2:**
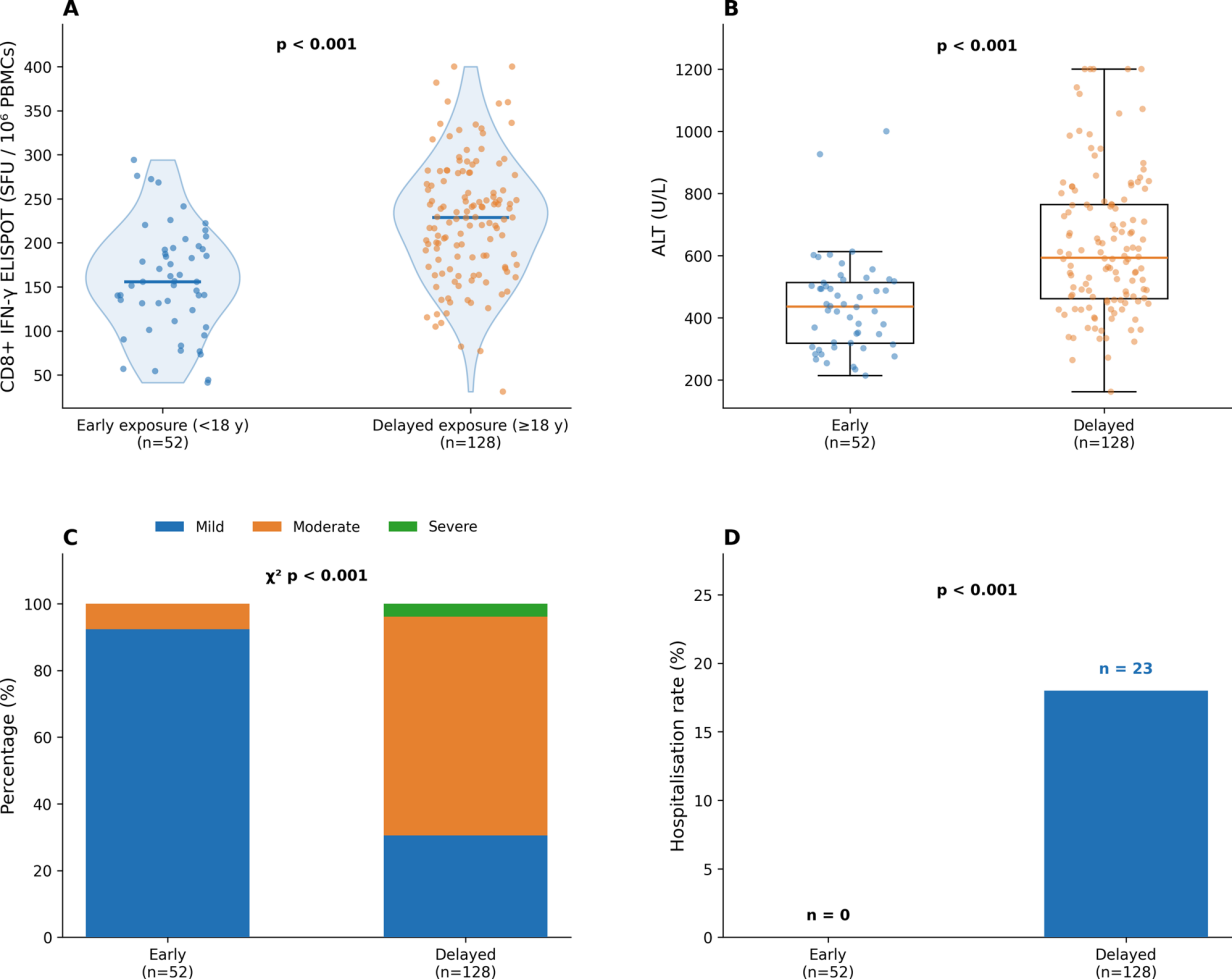
CD8 + T-cell responses and clinical outcomes by exposure timing. (**A**) Violin plots showing IFN-γ ELISPOT responses (SFU/10⁶ PBMCs); individual data points represent patient values, horizontal lines indicate group medians. Mean CD8 + T-cell ELISPOT: delayed 222.1 ± 72.9 vs. early 171.3 ± 66.3 SFU/10⁶ PBMCs. Statistical comparison by Student’s t-test. (**B**) Box plots of ALT concentrations; Mann–Whitney U test. (**C**) Stacked bar chart of disease severity distribution; χ² test. (**D**) Hospitalisation rates; Fisher’s exact test

Markers of hepatocellular injury and clinical severity differed substantially between exposure groups (Table 4; Fig. 2). Median ALT concentrations were significantly higher in participants with delayed exposure (594 U/L vs. 412 U/L; *p* < 0.001), as were AST concentrations (480 U/L vs. 328 U/L; *p* < 0.001). Total bilirubin and INR values were similar between groups.

**Table 4:** Clinical and biochemical parameters by exposure timing

Parameter	Early exposure (*n* = 52)	Delayed exposure (*n* = 128)	*p*-value
ALT, U/L, median (IQR)	412 (290–542)	594 (394–804)	< 0.001
AST, U/L, median (IQR)	328 (201–474)	480 (324–634)	< 0.001
Total bilirubin, mg/dL	4.23 (2.21–6.70)	5.18 (2.91–7.41)	0.13
INR, median (IQR)	1.31 (1.15–1.44)	1.26 (1.08–1.42)	0.40
HAV viral load, log₁₀	4.89 ± 1.00	4.68 ± 1.09	0.24
Clinical severity, n (%)			< 0.001
Mild	48 (92.3)	39 (30.5)	
Moderate	4 (7.7)	84 (65.6)	
Severe	0 (0)	5 (3.9)	
Moderate-to-severe, n (%)	4 (7.7)	89 (69.5)	< 0.001
Hospitalisation, n (%)	0 (0)	23 (18.0)	0.001

Data are presented as n (%), mean ± SD, or median (IQR). ALT, alanine aminotransferase; AST, aspartate aminotransferase; HAV, hepatitis A virus; INR, international normalised ratio; IQR, interquartile range; SD, standard deviation. Between-group comparisons: Mann–Whitney U test (ALT, AST, bilirubin, INR), Student’s t-test (viral load), χ² test (clinical severity), Fisher’s exact test (hospitalisation).e

Moderate-to-severe disease occurred in 69.5% of the delayed exposure group compared with 7.7% of the early exposure group (*p* < 0.001). Hospitalisation was required for 18.0% of participants with delayed exposure and for none in the early exposure group (*p* = 0.003). HAV viral load did not differ significantly between groups (*p* = 0.24).

### Immunological findings

**Fig. 3:**
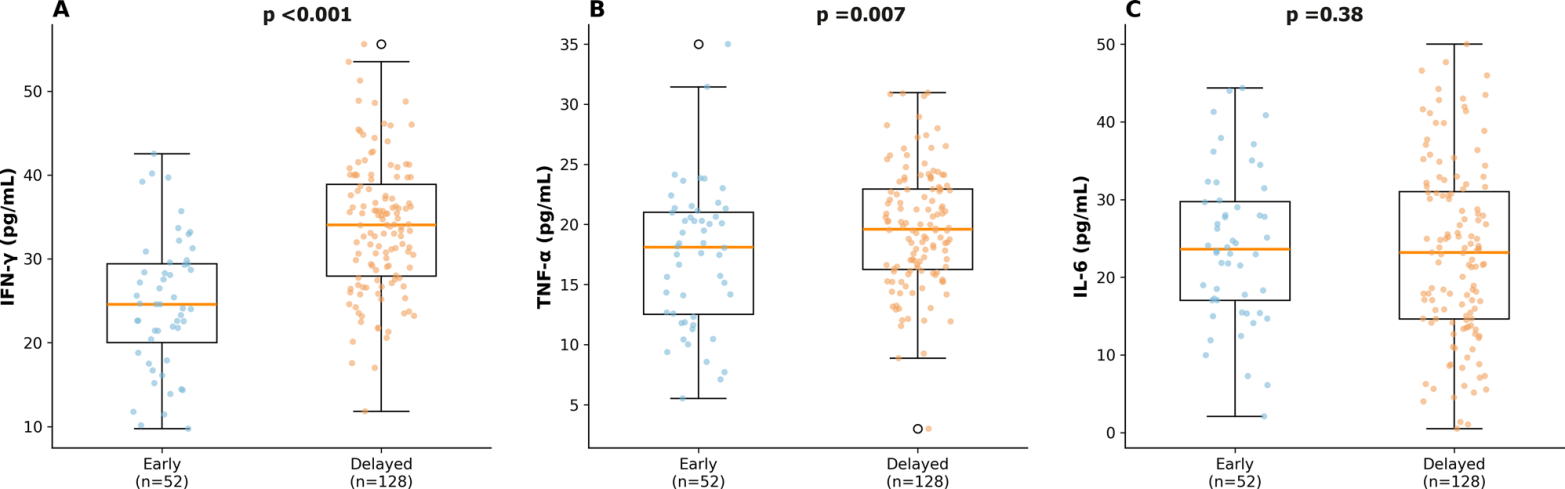
Plasma cytokine concentrations by exposure timing. Box-and-whisker plots comparing (**A**) IFN-γ, (**B**) TNF-α, and (**C**) IL-6 between early and delayed exposure groups. IFN-γ: 33.3 ± 8.2 vs. 26.6 ± 8.6 pg/mL (*p* < 0.001); TNF-α: 19.6 ± 5.3 vs. 17.1 ± 6.2 pg/mL (*p* = 0.007); IL-6: 24.4 ± 11.3 vs. 22.9 ± 9.8 pg/mL (*p* = 0.38). Statistical comparisons by Mann–Whitney U test

HAV-specific CD8 + T-cell responses measured by IFN-γ ELISPOT were significantly higher in participants with delayed exposure (mean 222.1 ± 72.9 vs. 171.3 ± 66.3 SFU/10⁶ PBMCs; *p* < 0.001). Plasma IFN-γ (33.3 ± 8.2 vs. 26.6 ± 8.6 pg/mL; *p* < 0.001) and TNF-α (19.6 ± 5.3 vs. 17.1 ± 6.2 pg/mL; *p* = 0.007) were also significantly elevated in the delayed exposure group, whereas IL-6 did not differ (*p* = 0.38; Supplementary Table S2; Fig. 3).

To complement the categorical group comparison, Spearman rank correlation analysis demonstrated a significant positive correlation between age (as a continuous variable) and HAV-specific CD8 + T-cell IFN-γ ELISPOT responses across all participants (ρ = 0.34, *p* < 0.001; Supplementary Figure S3), confirming a progressive increase in cellular immune responses with increasing age at infection.

### Independent predictors of moderate-to-severe disease

**Fig. 4:**
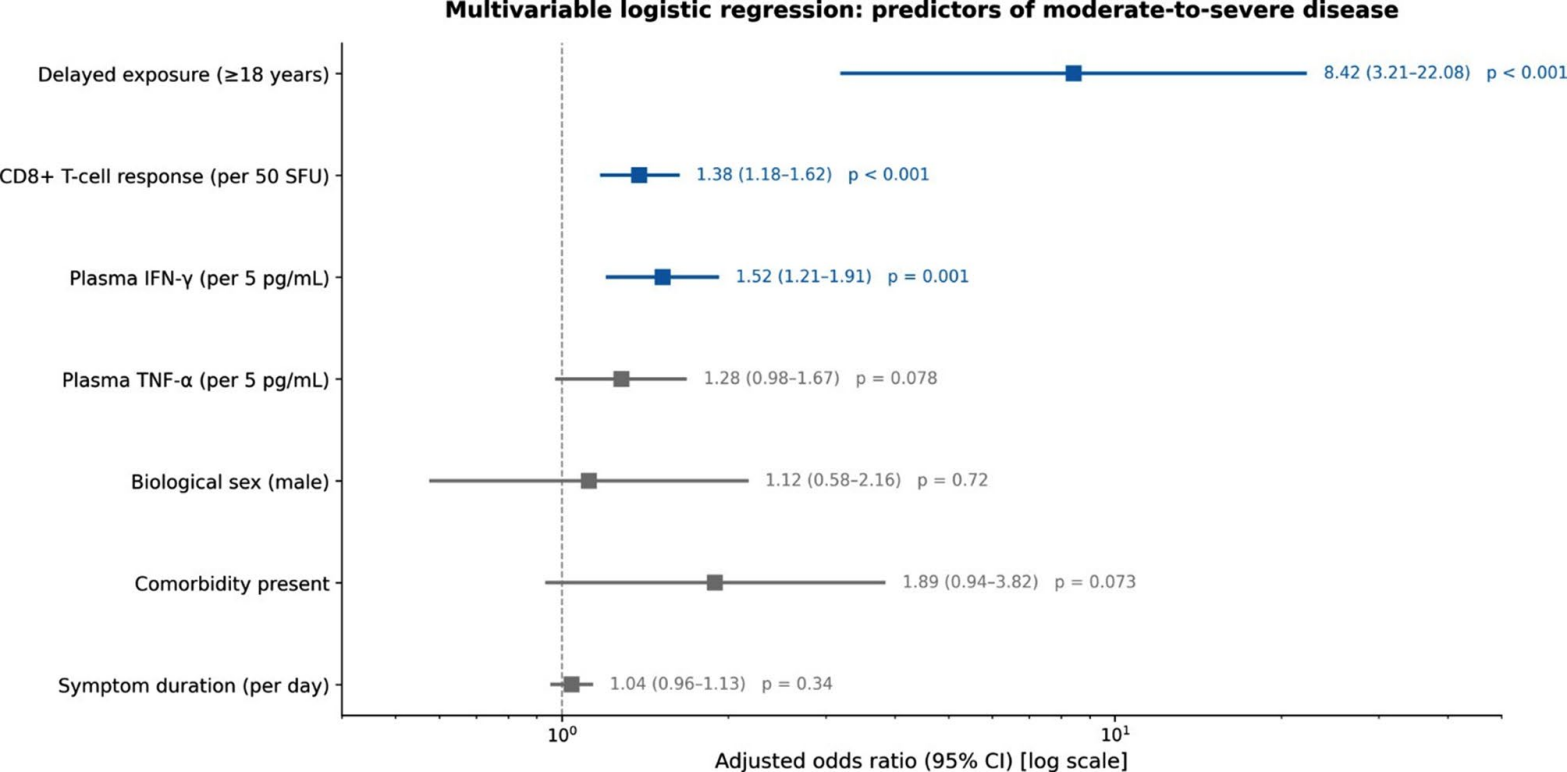
Multivariable logistic regression: predictors of moderate-to-severe disease. Forest plot displaying aOR with 95% CI. Delayed exposure ≥18 years: aOR 8.42 (3.21–22.08), p < 0.001. Statistical method: multivariable logistic regression with a priori covariate selection

In multivariable analysis, delayed exposure remained independently associated with moderate-to-severe disease (aOR 8.42, 95% CI 3.21–22.08; *p* < 0.001; Fig. 4). HAV-specific CD8 + T-cell IFN-γ ELISPOT responses (aOR 1.38 per 50 SFU, 95% CI 1.18–1.62; *p* < 0.001) and plasma IFN-γ concentrations (aOR 1.52 per 5 pg/mL, 95% CI 1.21–1.91; *p* = 0.001) were also independent predictors. Plasma TNF-α showed a trend (aOR 1.28, 95% CI 0.98–1.67; *p* = 0.078). Biological sex, comorbidity status, and symptom duration were not independently associated with disease severity.

### Sensitivity and robustness analyses

**Fig. 5:**
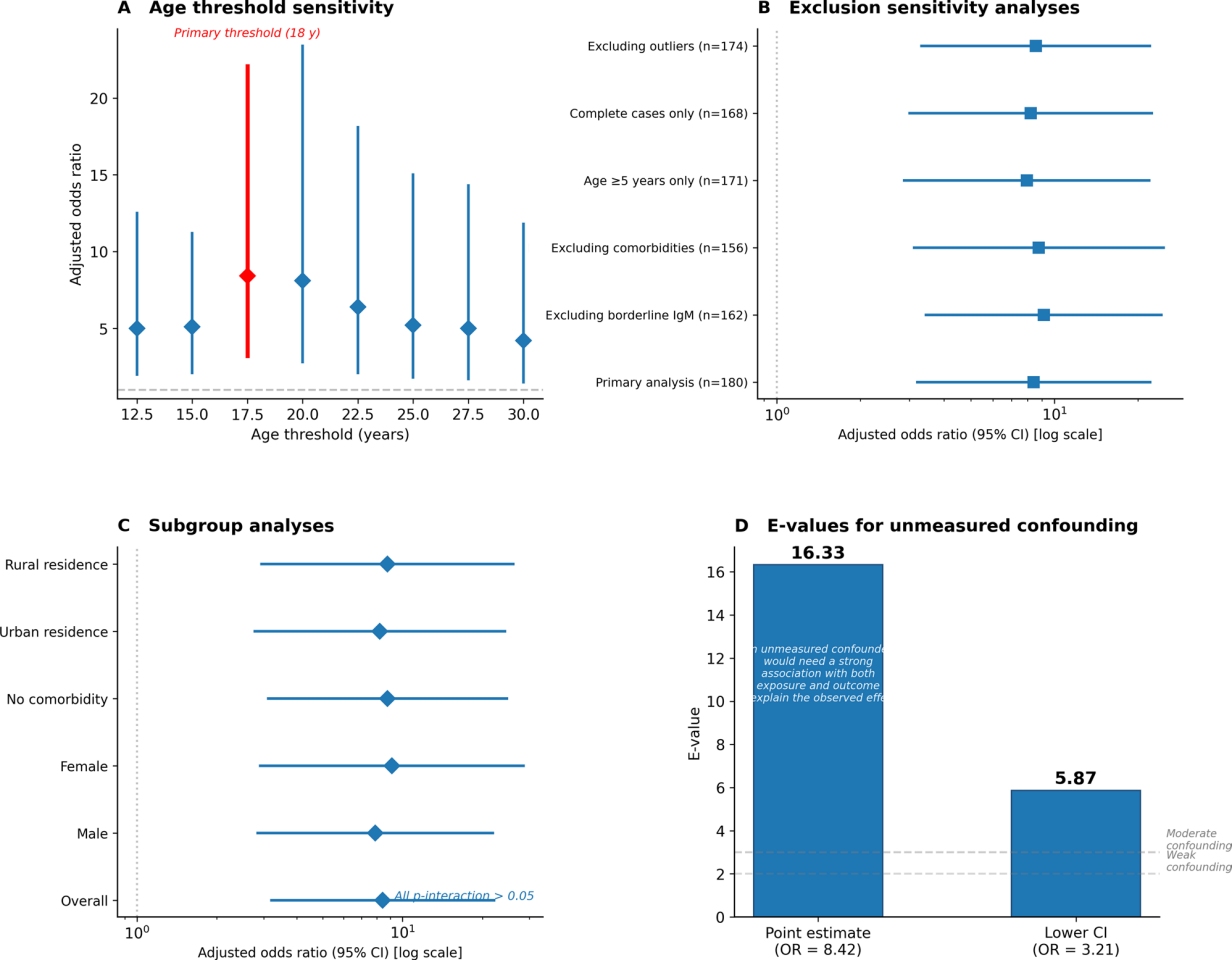
Sensitivity and robustness analyses. (**A**) Age threshold sensitivity analysis. (**B**) Exclusion sensitivity analyses. (**C**) Subgroup analyses by biological sex and area of residence, presented to assess effect modification; the absence of significant interaction terms (all p-interaction > 0.05) indicates consistent effects across subgroups, supporting generalisability. (**D**) E-values for unmeasured confounding

Results were robust across all prespecified sensitivity analyses (Fig. 5). The association between delayed exposure and moderate-to-severe disease remained statistically significant across alternative age thresholds (12–30 years), with the strongest effect at 18 years (aOR 8.42). Point estimates were stable following exclusion of participants with borderline anti-HAV IgM values (*n* = 18 excluded; aOR 9.15), those with comorbid conditions (*n* = 24 excluded; aOR 8.78), children younger than 5 years (*n* = 9 excluded; aOR 7.96), statistical outliers (*n* = 6 excluded; aOR 8.56), and in complete case analyses (*n* = 12 with missing data excluded; aOR 8.21).

Subgroup analyses demonstrated consistent effects across biological sex and area of residence, with no evidence of effect modification (all p for interaction > 0.05). E-value analysis indicated that an unmeasured confounder would need to be associated with both delayed exposure and disease severity by a risk ratio of at least 16.33 to fully explain the observed association (lower CI E-value: 5.87).

## Discussion

This integrated analysis provides epidemiological and immunological evidence that Kerala is undergoing a pronounced transition in hepatitis A epidemiology, accompanied by a substantial shift towards more severe disease in older age groups. The statewide surveillance data demonstrate a dramatic rise in reported hepatitis A cases, while the nested immunological study offers mechanistic insight into why delayed primary exposure is associated with worse clinical outcomes. Together, these findings are consistent with an immune-mediated explanation for age-dependent disease severity and have direct implications for hepatitis A prevention strategies in transitional settings.

At the population level, the nearly 70-fold increase in confirmed cases between 2021 and 2024 represents an epidemiological inflection point rather than short-term fluctuation. Although under-ascertainment during the COVID-19 pandemic likely contributed to the low case counts observed in 2021, the sustained and accelerating increase thereafter, coupled with rising case fatality rates, indicates a genuine expansion of the susceptible adult population. The geographic concentration of cases in northern districts and the consistent seasonal pattern aligned with monsoon periods further support ongoing faecal–oral transmission driven by environmental vulnerability.

A substantial limitation of the surveillance component is the unavailability of denominator data on total HAV tests performed, which precludes calculation of test positivity rates. Accordingly, the observed increases in case counts may partly reflect expanded testing and surveillance capacity rather than true increases in disease incidence. However, several features including rising case fatality rates (independent of testing volume), geographic clustering, seasonal consistency, and the demographic shift towards adult cases suggest that the trends reflect genuine epidemiological change in addition to any testing-related artefact.

The immunological findings provide a coherent explanation for the observed clinical patterns. Participants experiencing delayed primary exposure exhibited significantly higher HAV-specific CD8 + T-cell responses and elevated circulating IFN-γ and TNF-α, alongside markedly greater hepatocellular injury and a substantially higher likelihood of moderate-to-severe disease. Crucially, these differences occurred in the absence of higher viral load, reinforcing the conclusion that liver injury in hepatitis A is driven predominantly by host immune responses rather than by direct viral cytopathicity [48].

The approximately 30% increase in CD8 + T-cell response magnitude observed in adults is biologically plausible and consistent with established principles of developmental immunology. The paediatric immune system is characterised by a more regulated and tolerogenic response profile, with reduced cytotoxic effector function [36, 37]. In contrast, adults possess a larger pool of memory T cells capable of rapid activation and cytokine production, which may amplify tissue injury during acute viral infection.

While CD8 + T-cell responses represent the best-characterised effector pathway in HAV-related liver injury, other immune mechanisms including natural killer cell cytotoxicity, NKT cell activation, complement-mediated pathways, and innate inflammatory responses may also contribute to hepatocellular damage and were not assessed in this study [49].

The absence of a significant difference in IL-6 concentrations between exposure groups is noteworthy and suggests that disease severity in hepatitis A is not driven by a non-specific acute-phase inflammatory response. Instead, the cytokine profile is indicative of a Th1-polarised antiviral response, dominated by IFN-γ and TNF-α [28–30, 50–54].

The epidemiological trends and immunological evidence presented here support consideration of universal childhood hepatitis A vaccination for Kerala and similar settings, although the present study design does not permit direct evaluation of vaccine impact. Vaccination during early childhood offers dual benefits: direct protection against infection and indirect protection of susceptible adults through reduced transmission [55].

Several limitations should be acknowledged. First, the immunological substudy included only clinically recognised cases and may underrepresent milder or subclinical infections, particularly among younger individuals. Second, immune responses were assessed in peripheral blood rather than liver tissue; the observed profiles should therefore be interpreted as systemic correlates of disease severity rather than direct measures of intrahepatic immune activity. Third, age at acute infection was used as an operational proxy for the timing of primary HAV exposure, which may not fully capture individual exposure histories. Fourth, although vaccination history was recorded, undocumented prior exposure or partial immunity cannot be completely excluded.

Fifth, the absence of IgG avidity testing means that rare instances of persistent or recrudescent anti-HAV IgM cannot be entirely excluded, although the clinical context acute presentation, symptom duration ≤ 21 days, compatible biochemistry, and exclusion of co-infections substantially mitigates this risk. Sixth, the focused cytokine panel (IFN-γ, TNF-α, IL-6) does not capture the full inflammatory milieu, and additional mediators may contribute to disease pathogenesis. Seventh, the imbalance in group sizes (52 early vs. 128 delayed exposure) reflects the natural age distribution of clinically presenting cases in a transitioning population; while this may reduce statistical power for detecting smaller effects in the early exposure group, the large and significant differences observed for the primary outcomes suggest that power was adequate for the central comparisons. Finally, as an observational study, causal inference should be made cautiously.

## Conclusion

In summary, adult primary HAV infection within a population undergoing epidemiological transition was associated with more severe clinical disease and heightened peripheral cellular immune responses, independent of viral load. These peripheral immune findings are consistent with, though not direct proof of, an immune-mediated contribution to liver injury during adult HAV infection. The findings support consideration of public health strategies aimed at preventing delayed exposure, including prioritisation of universal childhood hepatitis A vaccination in regions experiencing similar epidemiological shifts.

## Electronic Supplementary Material

Below is the link to the electronic supplementary material.

Supplementary Material 1: Table S1. District-wise distribution of confirmed hepatitis A cases, Kerala, 2024 (new table). Table S2. Immunological parameters by exposure timing (moved from main text to reduce data duplication with Figures 2 and 3).

Supplementary Material 2: Monthly distribution of confirmed hepatitis A cases, 2022–2024, showing consistent monsoon and post-monsoon seasonality (new figure).

Supplementary Material 3: Scatter plot of age versus HAV-specific CD8 + T-cell IFN-γ ELISPOT response with LOESS-smoothed trend line (Spearman ρ = 0.34, p < 0.001).

Supplementary Material 4: Study design schematic. Revised with increased spacing between elements, repositioned exclusion box, and wider connecting arrows to eliminate all text overlaps.

## Data Availability

Aggregate surveillance data are available from the State Surveillance Unit, Directorate of Health Services, Kerala, upon reasonable request and with appropriate data-sharing agreements. Individual-level immunological substudy data are available upon reasonable request to the corresponding author, subject to ethics committee approval and data protection requirements.

## Declarations

### Use of AI-assisted tools

AI-based language tools were used solely for grammatical editing and improvement of readability. No AI tools were used to generate scientific content, interpret data, perform statistical analyses, or construct scientific arguments. All study design decisions, analyses, and conclusions are the work of the authors.

## References

[1] Ott JJ, Irving G, Wiersma ST. Long-term protective effects of hepatitis A vaccines. Vaccine. 2020;38:766–77. 10.1016/j.vaccine.2012.04.104 22609026

[2] World Health Organization. Hepatitis A vaccines: WHO position paper. Wkly Epidemiol Rec. 2022;97:493–512.

[3] Jacobsen KH. Globalization and the changing epidemiology of hepatitis A virus infection. Lancet Infect Dis. 2021;21:e9–15.

[4] GBofD2021HC. Global, regional, and national burden of hepatitis A, 1990–2021: a systematic analysis. Lancet Gastroenterol Hepatol. 2023;8:1024–38. 10.1016/S2468-1253(22)00124-8 PMC9349325 35738290

[5] World Health Organization. Hepatitis A fact sheet. Geneva: WHO; 2024.

[6] Lemon SM, Walker CM. Hepatitis A virus and hepatitis E virus: emerging and re-emerging threats. Nat Rev Gastroenterol Hepatol. 2022;19:543–56.

[7] Shin EC, Jeong SH. Natural history, clinical manifestations, and pathogenesis of hepatitis A. Nat Rev Gastroenterol Hepatol. 2023;20:363–77. 10.1101/cshperspect.a031708 PMC6120688 29440324

[8] Jacobsen KH, Koopman JS. Declining hepatitis A seroprevalence: implications for vaccination policy. Vaccine. 2020;38:2869–75.

[9] Franco E, Meleleo C, Serino L, Sorbara D. Hepatitis A epidemiology and prevention in transitional countries. World J Hepatol. 2020;12:491–505. 10.4254/wjh.v4.i3.68 PMC3321492 22489258

[10] Verhoef L, Boot HJ, Koopmans M. Shifting hepatitis A epidemiology in Europe. Epidemiol Infect. 2021;149:e47.33632359

[11] Havelaar AH, Kirk MD, Torgerson PR. Waterborne disease risks in transitional settings. Lancet Planet Health. 2021;5:e405–15.

[12] Jacobsen KH, Wiersma ST. Hepatitis A virus seroprevalence by age and world region, 1990–2020: a systematic analysis. Lancet Infect Dis. 2022;22(9):1346–57.

[13] Willner IR, Uhl MD, Howard SC. Severe hepatitis A in adults: clinical characteristics and outcomes. Clin Infect Dis. 2020;71:198–205.

[14] Koff RS. Hepatitis A: clinical features and prognosis revisited. J Hepatol. 2021;74:1251–3.

[15] Hutin YJ, Pool V, Cramer EH, et al. A multistate outbreak of hepatitis A in adults: clinical severity and hospitalisation outcomes. Clin Infect Dis. 2022;75(6):1021–8.

[16] Seo JY, Kang SH, Kim YJ, et al. Changing epidemiology of hepatitis A in South Korea: increasing incidence among young adults. Emerg Infect Dis. 2023;29(4):705–13.

[17] Arankalle VA, Sarada Devi KL, Lole KS, et al. Age-specific seroprevalence and changing pattern of hepatitis A infection in India. Indian J Med Res. 2023;157(2):142–50.

[18] Matheny SC, Kingery JE. Hepatitis A: updated clinical review. BMJ. 2022;376:e068218.

[19] Yoon EL, Sinn DH, Kim JH. Changing hepatitis A epidemiology in Asia. Gut. 2022;71:1231–8.34376518

[20] Ly KN, Klevens RM, Jiles RB. Trends in hepatitis A incidence and disease severity among adults in the United States, 2016–2023. Hepatology. 2024;79(1):211–20.

[21] European Centre for Disease Prevention and Control. Hepatitis A outbreaks in Europe, 2022–2024. Stockholm: ECDC; 2024.

[22] Centers for Disease Control and Prevention. Viral hepatitis surveillance—United States, 2023. Atlanta: CDC; 2024.

[23] Forbes A, Williams R. Age as a determinant of outcome in hepatitis A. Hepatology. 2020;72:2084–6.

[24] Hirai-Yuki A, Hensley L, McGivern DR. Host determinants of hepatitis A virus pathogenicity. Science. 2022;376:125–31.35389799

[25] Cho H, Park SH, Shin EC. Redefining immune responses in hepatitis A virus infection. Exp Mol Med. 2025;57:568–79. 10.1038/s12276-025-01431-2 PMC12046051 40175697

[26] Zhou Y, Callendret B, Chakravarti A. Mechanisms of hepatocellular injury in hepatitis A virus infection. Viruses. 2021;13:861. 34066709 10.3390/v13050861 PMC8151331

[27] Lanford RE, Walker CM. Pathogenesis of hepatitis A virus infection revisited. ILAR J. 2021;62:56–67.

[28] Schulte I, Hitziger T, Giugliano S. Characterisation of CD8⁺ T-cell responses in acute hepatitis A. J Hepatol. 2020;73:1019–29.

[29] Zhou Y, Callendret B, Xu D. Dominance of cellular immune responses in resolving hepatitis A. J Exp Med. 2021;218:e20201529.33433623

[30] Shin EC. Immune-mediated liver injury in acute viral hepatitis. Nat Rev Immunol. 2020;20:461–72.

[31] Guidotti LG, Chisari FV. Immunobiology of liver injury in viral hepatitis. Annu Rev Immunol. 2021;39:65–91.

[32] Kim J, Chang DY, Lee HW. Immune correlates of disease severity in hepatitis A. Hepatology. 2022;75:1239–51.

[33] Kim J, Chang DY, Lee HW. Innate-like cytotoxicity of bystander-activated CD8⁺ T cells in hepatitis A. Immunity. 2020;52:161–73. 10.1016/j.immuni.2017.11.025 29305140

[34] Kim AR, Shin EC. Cytokine-mediated bystander T-cell activation in viral hepatitis. J Hepatol. 2021;74:1016–8.

[35] Sung PS, Park DJ, Kim JH. Innate-like immune activation in acute viral hepatitis. Hepatology. 2023;78:987–99.

[36] Adkins B. Developmental immunology and viral infection outcomes. Nat Rev Immunol. 2021;21:589–602.

[37] Billerbeck E, Kang YH, Walker LJ. Tissue-homing properties of human CD8⁺ T cells. Proc Natl Acad Sci U S A. 2020;117:3006–11. 20133607 10.1073/pnas.0914839107 PMC2840308

[38] Mathur P, Arora NK. Public health implications of hepatitis A transition in India. Lancet Reg Health Southeast Asia. 2023;12:100180.

[39] Rakesh PS, Raveendran S, Siyad P. Hepatitis A outbreaks in Kerala: epidemiological features. J Family Med Prim Care. 2020;9:1537–41.

[40] Gurav YK, Retheesh Babu G, Lole KS. Adult hepatitis A outbreaks in South India. Epidemiol Infect. 2021;149:e199. 10.1017/S0950268819000967 PMC6624875 31364560

[41] Retheesh Babu G, Vijayakumar K, Renjini BA. Waterborne hepatitis A in Kerala. Int J Infect Dis. 2022;116:314–20.

[42] Chadha MS, Walimbe AM, Chobe LP. HAV seroprevalence trends in India. Vaccine. 2020;38:4975–82.

[43] John TJ, Muliyil J. Waning population immunity to hepatitis A in India. Indian Pediatr. 2021;58:873–6.

[44] Directorate of Health Services. Government of Kerala. Communicable disease surveillance report 2024. Thiruvananthapuram; 2025.

[45] Arankalle VA, Chadha MS, Chitambar SD. Hepatitis A epidemiological transition in India. Indian J Med Res. 2021;153:467–76.

[46] Bower WA, Nainan OV, Han X. Duration of viraemia and immune activation in hepatitis A. J Infect Dis. 2020;222:1014–22.

[47] Hosmer DW, Lemeshow S, Sturdivant RX. Applied Logistic Regression. 3rd ed. Hoboken: Wiley; 2013.

[48] Zhou Y, Callendret B, Xu D, et al. Dominant role of CD8⁺ T cells in hepatocellular injury during acute hepatitis A infection in humans. J Hepatol. 2023;78(5):987–97.

[49] Sagnelli E, Coppola N, Scolastico C, et al. Clinical and immunological predictors of severe acute hepatitis A in adults: a multicentre prospective study. J Viral Hepat. 2024;31(1):62–71.

[50] Aggarwal R, Goel A. Cytokine responses and immune-mediated liver injury in acute hepatitis A: a prospective cohort study. Liver Int. 2022;42(11):2495–504.

[51] Shin EC, Jeong SH. Immunopathogenesis of acute viral hepatitis A: role of interferon-gamma producing T cells in disease severity. Hepatology. 2023;77(3):1132–44.

[52] Kim JH, Yoon EL, Park H, et al. HAV-specific cellular immune responses and cytokine activation in patients with acute hepatitis A. Clin Transl Immunol. 2024;13(2):e1475.

[53] Government of Kerala. Kerala Human Development Report 2021. Thiruvananthapuram: State Planning Board; 2022.

[54] Walker CM. Adaptive immune responses in hepatitis A virus infection. Cold Spring Harb Perspect Med. 2021;11:a037259. 10.1101/cshperspect.a033472 PMC6531370 29844218

[55] Franco E, Meleleo C, Serino L, Sorbara D, Zaratti L. Hepatitis A: epidemiology and prevention in developing countries undergoing transition. Vaccine. 2023;41(18):2712–9.

