# Supplementary material for: Immunological consequences of delayed exposure to hepatitis A virus: evidence consistent with CD8+ T-cell–mediated immunopathology
Source: BMC Infect Dis. 2026 Apr 11;26:888. doi: 10.1186/s12879-026-13193-x (PMC13137650; doi:10.1186/s12879-026-13193-x)
Supplement: Supplementary file 1 — Supplementary Material 1: Table S1. District-wise distribution of confirmed hepatitis A cases, Kerala, 2024 (new table). Table S2. Immunological parameters by exposure timing (moved from main text to reduce data duplication with Figures 2 and 3). [file 12879_2026_13193_MOESM1_ESM.docx]

**Supplementary Table S1. District-wise distribution of confirmed hepatitis A cases, Kerala, 2024**

| **District** | **Confirmed cases** | **Deaths** | **CFR (%)** |
| --- | --- | --- | --- |
| Malappuram | 3,412 | 34 | 1.00 |
| Kozhikode | 1,287 | 18 | 1.40 |
| Kannur | 498 | 8 | 1.61 |
| Kasaragod | 421 | 3 | 0.71 |
| Palakkad | 389 | 5 | 1.29 |
| Thrissur | 367 | 4 | 1.09 |
| Ernakulam | 312 | 6 | 1.92 |
| Thiruvananthapuram | 298 | 3 | 1.01 |
| Pathanamthitta | 245 | 2 | 0.82 |
| Kottayam | 198 | 2 | 1.01 |
| Other districts | 540 | 4 | 0.74 |
| **Total** | **7,967** | **89** | **1.12** |

*CFR, case fatality rate. Other districts include Alappuzha, Idukki, Kollam, and Wayanad. Source: Integrated Disease Surveillance Programme, State Surveillance Unit, Directorate of Health Services, Government of Kerala. The geographic concentration of cases in 2024 remained consistent with patterns observed in 2023 (Table 2), with Malappuram and Kozhikode districts continuing to bear a disproportionate share of the disease burden.*

**Supplementary Table S2 Immunological parameters by exposure timing**

| **Parameter** | **Early exposure (n = 52)** | **Delayed exposure (n = 128)** | **p-value** |
| --- | --- | --- | --- |
| CD8⁺ T-cell IFN-γ ELISPOT (SFU/10⁶ PBMCs) | 171.3 ± 66.3 | 222.1 ± 72.9 | <0.001 |
| Plasma IFN-γ (pg/mL) | 26.6 ± 8.6 | 33.3 ± 8.2 | <0.001 |
| Plasma TNF-α (pg/mL) | 17.1 ± 6.2 | 19.6 ± 5.3 | 0.007 |
| Plasma IL-6 (pg/mL) | 22.9 ± 9.8 | 24.4 ± 11.3 | 0.38 |

*Data are presented as mean ± SD. ELISPOT, enzyme-linked immunospot; IFN-γ, interferon-gamma; IL-6, interleukin-6; PBMCs, peripheral blood mononuclear cells; SD, standard deviation; SFU, spot-forming units; TNF-α, tumour necrosis factor-alpha. Between-group comparisons: Student’s t-test (ELISPOT), Mann–Whitney U test (plasma cytokines). This table was moved from the main text to supplem*

**Supplementary Table S1.** District-wise distribution of confirmed hepatitis A cases, Kerala, 2024.

**Supplementary Table S2 .** Immunological parameters by exposure timing.
