# Supplementary figures and images for: Immunological consequences of delayed exposure to hepatitis A virus: evidence consistent with CD8+ T-cell–mediated immunopathology
Source: BMC Infect Dis. 2026 Apr 11;26:888. doi: 10.1186/s12879-026-13193-x (PMC13137650; doi:10.1186/s12879-026-13193-x)

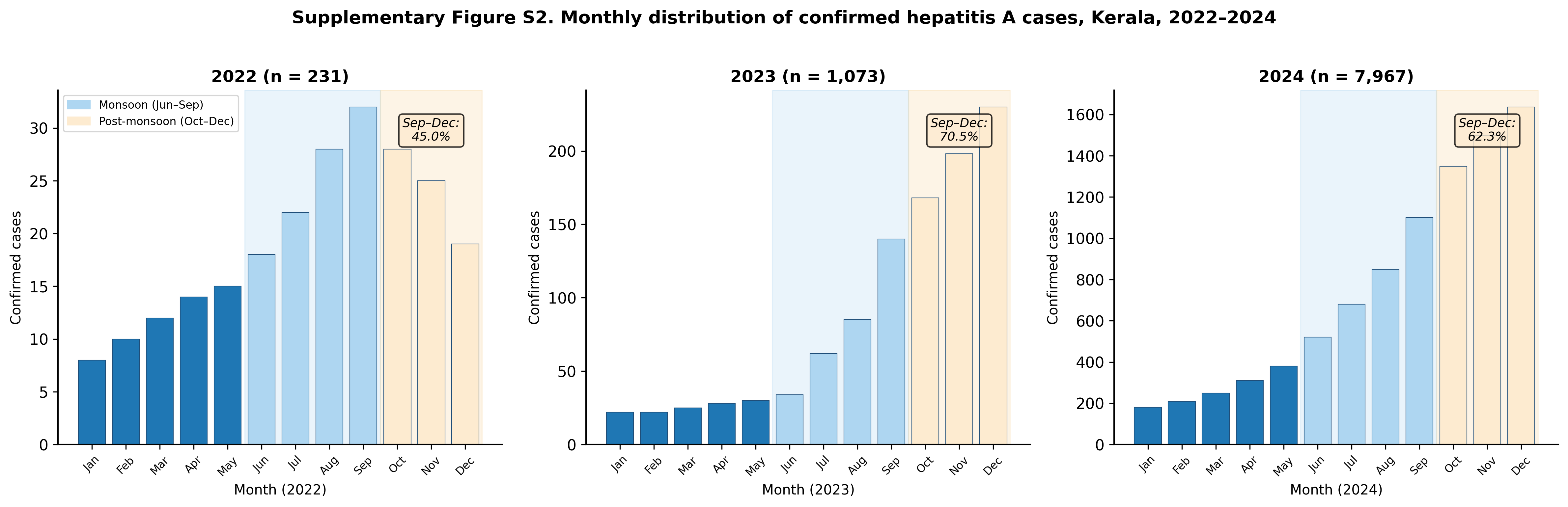

Supplement: Supplementary file 2 — Supplementary Material 2: Monthly distribution of confirmed hepatitis A cases, 2022–2024, showing consistent monsoon and post-monsoon seasonality (new figure). [file 12879_2026_13193_MOESM2_ESM.png]

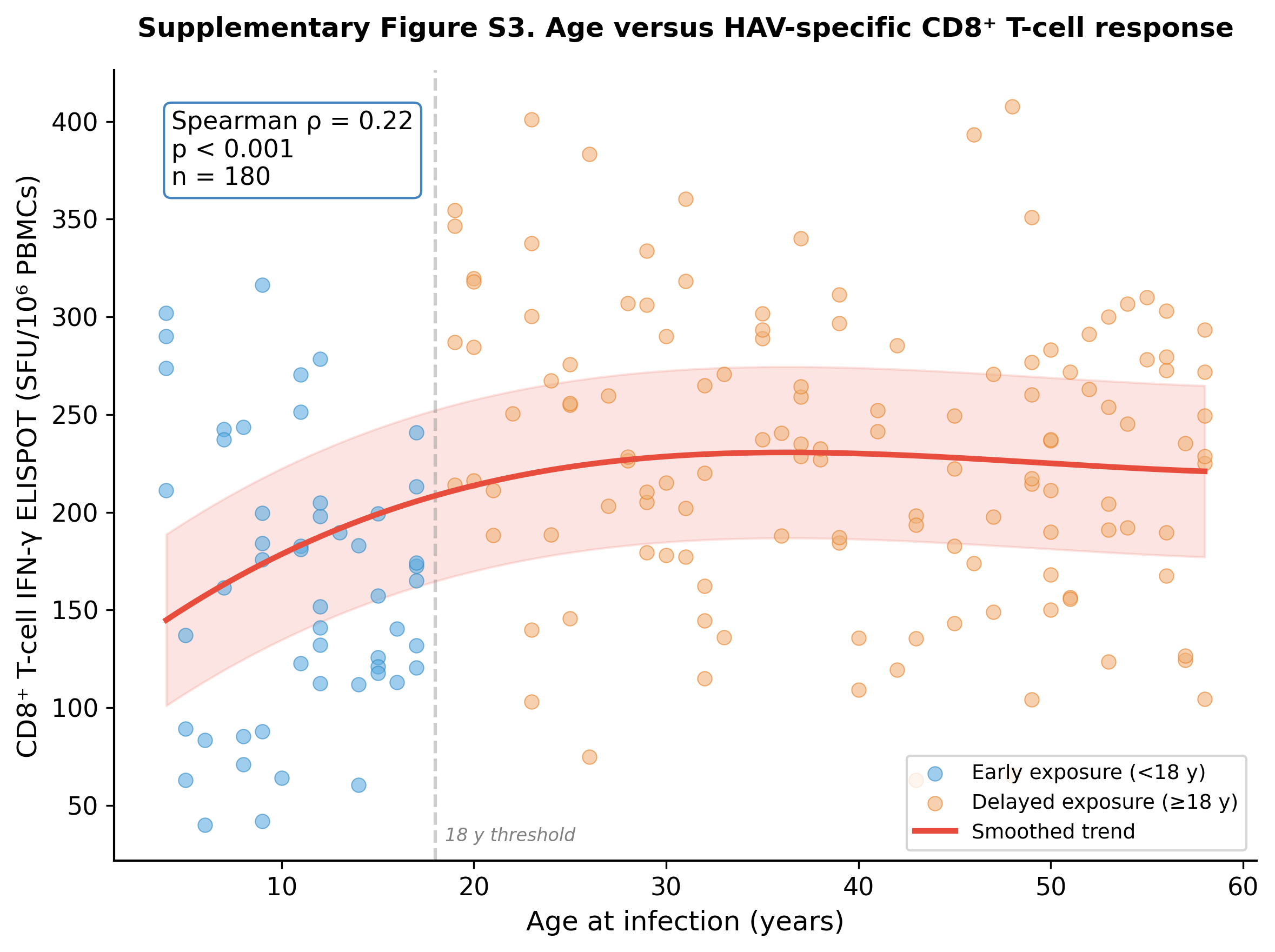

Supplement: Supplementary file 3 — Supplementary Material 3: Scatter plot of age versus HAV-specific CD8 + T-cell IFN-γ ELISPOT response with LOESS-smoothed trend line (Spearman ρ = 0.34, p < 0.001). [file 12879_2026_13193_MOESM3_ESM.png]

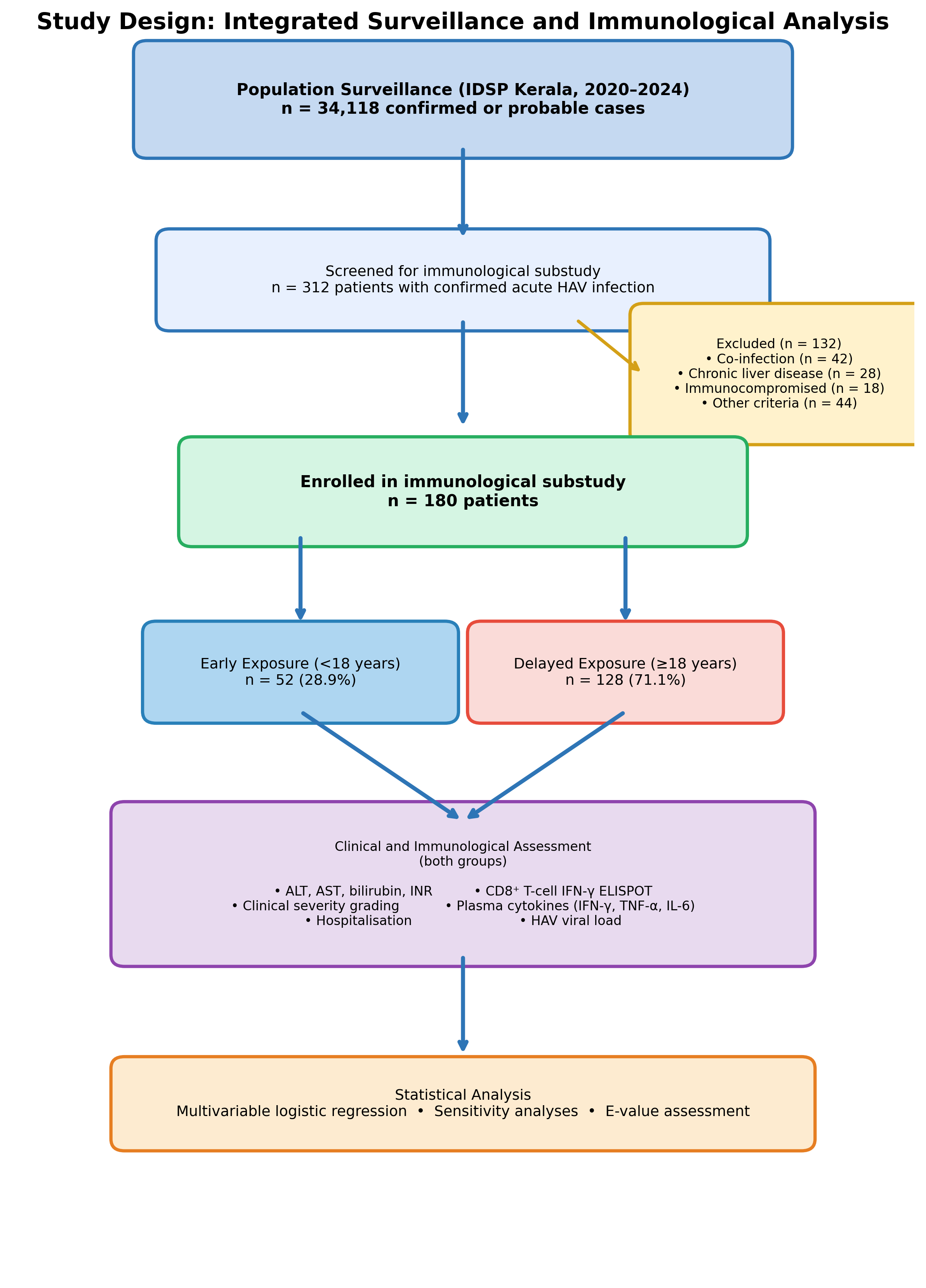

Supplement: Supplementary file 4 — Supplementary Material 4: Study design schematic. Revised with increased spacing between elements, repositioned exclusion box, and wider connecting arrows to eliminate all text overlaps. [file 12879_2026_13193_MOESM4_ESM.png]
